# Mating Behaviour in Laevicaudatan Clam Shrimp (Crustacea, Branchiopoda) and Functional Morphology of Male Claspers in a Phylogenetic Context: A Video-Based Analysis

**DOI:** 10.1371/journal.pone.0084021

**Published:** 2014-01-02

**Authors:** Zandra M. S. Sigvardt, Jørgen Olesen

**Affiliations:** Natural History Museum of Denmark, Copenhagen, Denmark; Sars International Centre for Marine Molecular Biology, Norway

## Abstract

Clam shrimps are freshwater branchiopod crustaceans which often present complicated breeding systems including asexual reproduction (parthenogenesis) and mixed mating systems (in androdioecious species both selfing and outcrossing occurs due to the co-presence of hermaphrodites and males). Reproductive patterns of Spinicaudata, which contains most clam shrimp species, have received much attention. Another group of clam shrimps, Laevicaudata, which holds a key position in branchiopod phylogeny, has practically not been studied. As a part of the mating process, males clasp to the carapace margin of the females with a pair (or two pairs) of anterior trunk limbs modified as claspers. Previous studies have shown that clasper morphology is important in a phylogenetic context, and that some parts of the claspers in Spinicaudata and Laevicaudata may have undergone a remarkable parallel evolution. Here we have used video microscopy to study aspects of the mating behaviour, egg extrusion, and fertilization in *Lynceus brachyurus* (Laevicaudata). It is shown that fertilization is likely to be external and that the peculiar tri-lobed lateral lamellae of female's hind body assist in guiding the egg mass to the exopodal egg carriers where they are collected by their distal setation. The functional morphology of the male claspers was studied in detail by close-up video recordings. The movable “finger” of the clasper bends around the female's carapace edge and serves to hold the female during mating. The larger palp grasps around the female carapace margin in a way very similar to the movable “finger”, possibly indirectly providing sensory input on the “finger” position. A brief comparative study of the claspers of a spinicaudatan clam shrimp showed both similarities and differences to the laevicaudatan claspers. The presence of two pairs of claspers in Spinicaudata seems to give males a better hold of the female which may play a role during extended mate guarding.

## Introduction

Clam shrimps are freshwater crustaceans with a hinged bivalved carapace giving them a superficial resemblance to bivalves (molluscs). About 500 species are known world wide, but they are mostly rare with a scattered distribution and nearly always inhabit small temporary aquatic systems [1]. Despite many similarities in morphology (e.g., bivalved carapace), feeding mode (e.g., filtratory), and in lifestyle in general, recent phylogenetic studies have divided clam shrimps in three separate groups, Spinicaudata, Laevicaudata, and Cyclestherida (reviewed in [2]). The monotypic Cyclestherida is sister group to the diverse and evolutionarily miniaturized Cladocera (waterfleas), with Spinicaudata as their likely sister group [2], [3], [4], [5], [6], but the precise position of the remaining group of clam shrimps, the Laevicaudata, has proved more difficult to establish.

**Figure 1:**
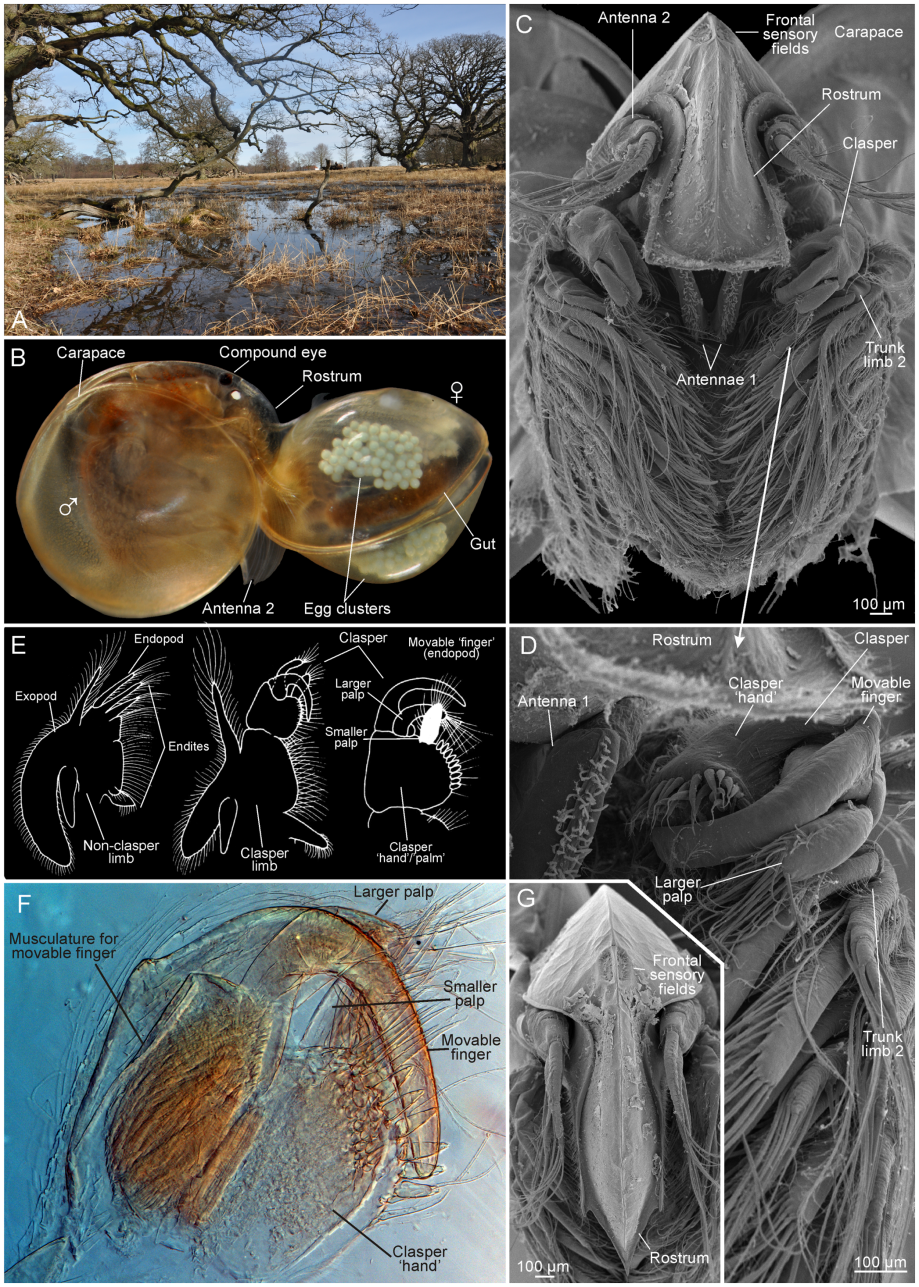
The clam shrimp *Lynceus brachyurus* (Laevicaudata); morphological overview and sampling locality. A, one of the two collecting sites where material has been collected. B, male clasping female during mating. C, male with carapace removed showing first pairs of trunk limbs modified as claspers. D, higher magnification of C showing right side clasper. E, schematic drawings of clasper limb and non-clasper limb (redrawn from Olesen *et al.* 1996). F, clasper of right side in LM showing musculature of movable finger. G, female rostrum.

Clam shrimp and cladoceran males have clasping structures on the first pairs (two first pairs in Spinicaudata) of trunk limbs. It is well-known that these structures are used by the male to clasp the edge of the female's carapace during pairing, sometimes for many hours (e.g., *Eulimnadia texana* Packard, 1871, see [7]). Among other branchiopods, the males of anostracans and the Devonian *Lepidocaris rhyniensis* have appendages modified for clasping, but these are non-homologous as other limbs are involved (e.g, [8], [9]). Clasping structures of the trunk limbs of clam shrimps and water fleas have played an important role in establishing a morphology-based phylogeny of the Branchiopoda, but the homologies between major taxa of the various clasper parts have long been discussed [4], [10], [11], [12], [13]. The presence of clasping devices on the anterior trunk limbs of all ‘bivalved branchiopods’ (clam shrimps and cladocerans) is generally seen as an evolutionary novelty in this lineage [4], [13], [15]. The claspers of all clam shrimps (Spinicaudata, Cyclestherida, and Laevicaudata) are indeed very similar and consist essentially of four parts: an inflated limb part (the clasper ‘hand’ or ‘palm’) carrying three structures, a so-called movable ‘finger’ and two additional projections termed ‘palps’ (a smaller and a larger) (Fig. 1E, F). Cladoceran claspers are more diverse [16]. Despite these profound similarities between clam shrimp claspers, the precise homologies are still being debated. The exact homologies of the various clasper parts has been studied (or discussed) by various authors [10], [13], [14], [17], and, despite the similarities, it has been suggested that parts of the claspers have have been derived independently in Spinicaudata and Laevicaudata (the ‘hand’ and the palps). No information is available on the details of how claspers in clam shrimps operate during clasping to facilitate mating, which would be of evolutionary interest in the light of the possible non-homologous status of certain clasper parts.

It is known that the male uses the claspers for clinging to the female during mate guarding and mating, but which structures of the clasper (the ‘hand’, the movable ‘finger’, or the two palps) play a role in this process? And what is the exact nature of this role? A central question is: Are the function of the various clasper parts (e.g., the ‘hand’ and the palps) in different groups of clam shrimps the same despite being derived in at least partly different ways? Or is this partly independent derivation reflected in the function? Information on this may offer important insight into the mechanisms behind parallel (convergent) evolution in general, and into branchiopod evolution in particular. As a first step in elucidating these questions we here provide a study of the functional morphology of the male claspers in a laevicaudatan clam shrimp (*Lynceus brachyurus*) using close-up video recordings combined with morphological studies. A brief morphological study of the claspers of a spinicaudatan clam shrimp (*Cyzicus californicus*) is included for comparison. We also include more general observations of the mating in *L. brachyurus*, such as on the male's ‘brushing behaviour’ and its relation to the timing of female egg extrusion, since this has never been reported in detail for the phylogenetically pivotal Laevicaudata.

## Materials and Methods

**Figure 2:**
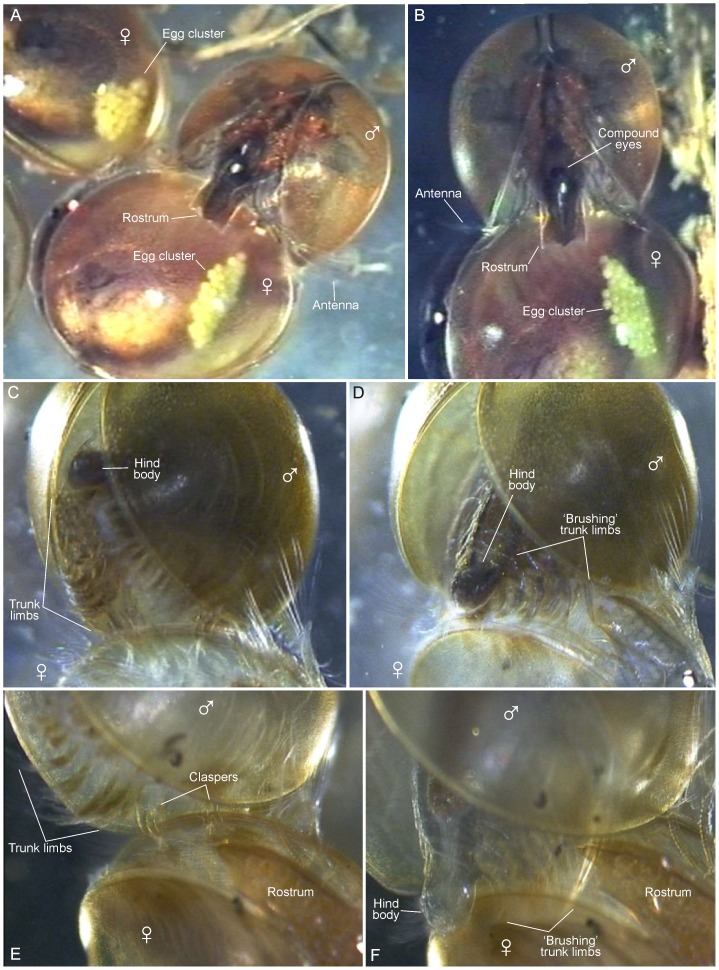
Male-female mating in *Lynceus brachyurus* (Laevicaudata) based on frames grabbed from video recordings. A, B, male clasping ventral carapace edge of female. C, male clasped to female with hind body outside female's carapace valves. D, male bending hind body towards female's carapace valves while trunk limbs make brushing movements. E, male clasped to female with hind body outside female's carapace valves. F, male bending hind body close to female's carapace valves; male's trunk limbs are inserted between valves and assist spreading female's carapace valves when stretching body slightly.

**Figure 3:**
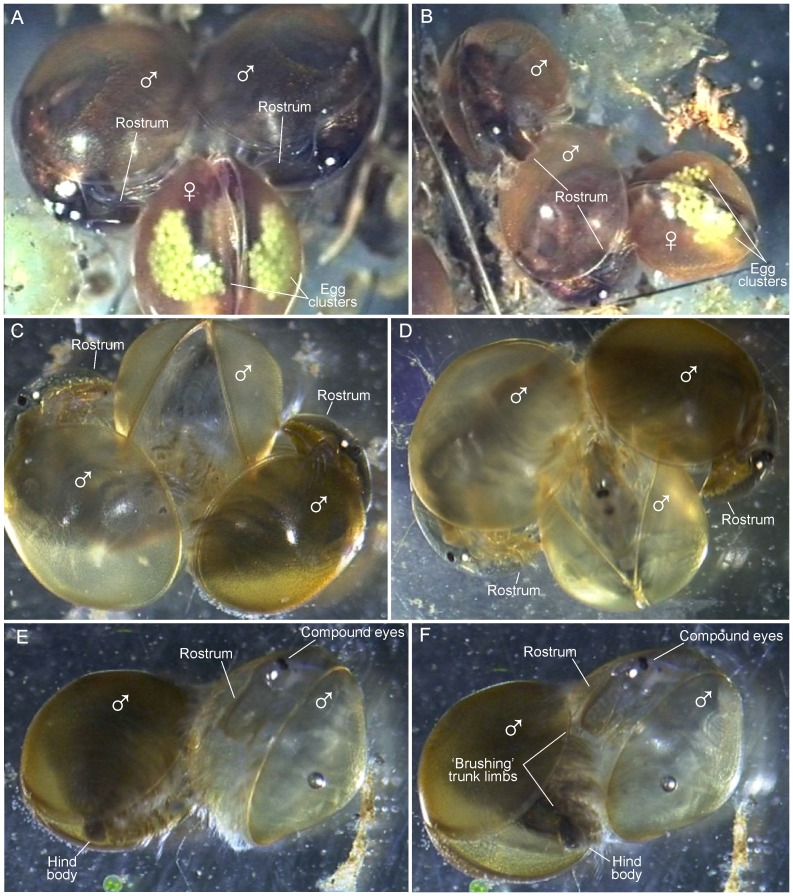
Male-male-female mating interactions in *Lynceus brachyurus* (Laevicaudata) based on frames grabbed from video recordings. A, two males clasping the same female. B, male clasping another male which is clasping a female (‘train’ formation). C, two males clasping another male. D, two males clasping another male. E, male clasping another male prior to ‘brushing’. F, male clasping another male and bending its hind body and ‘brushing’ with its trunk limbs.

**Figure 4:**
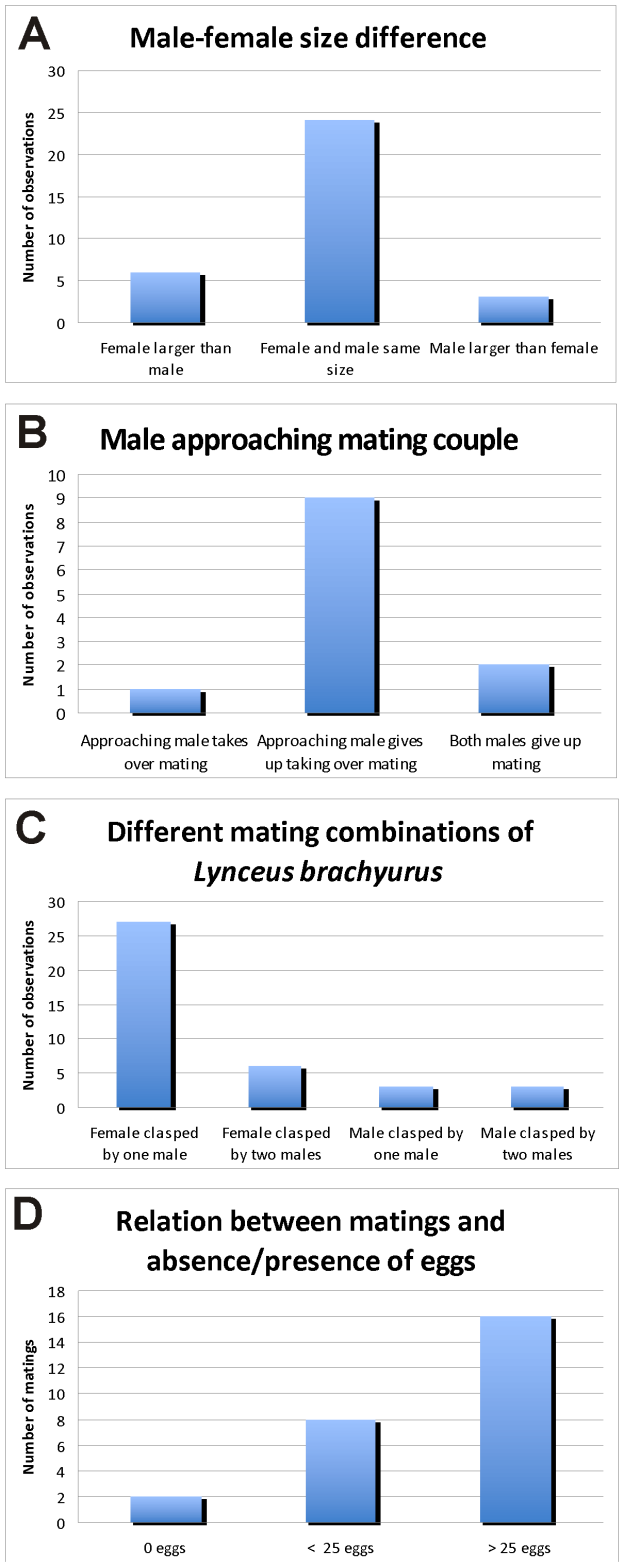
Mating behaviour in the clam shrimp *Lynceus brachyurus* (Laevicaudata). A, size relation between male and female during mating. B, effect of male approaching an already mating couple. C, Different mating combinations between males and females. D, number of eggs present during mating.

**Figure 5:**
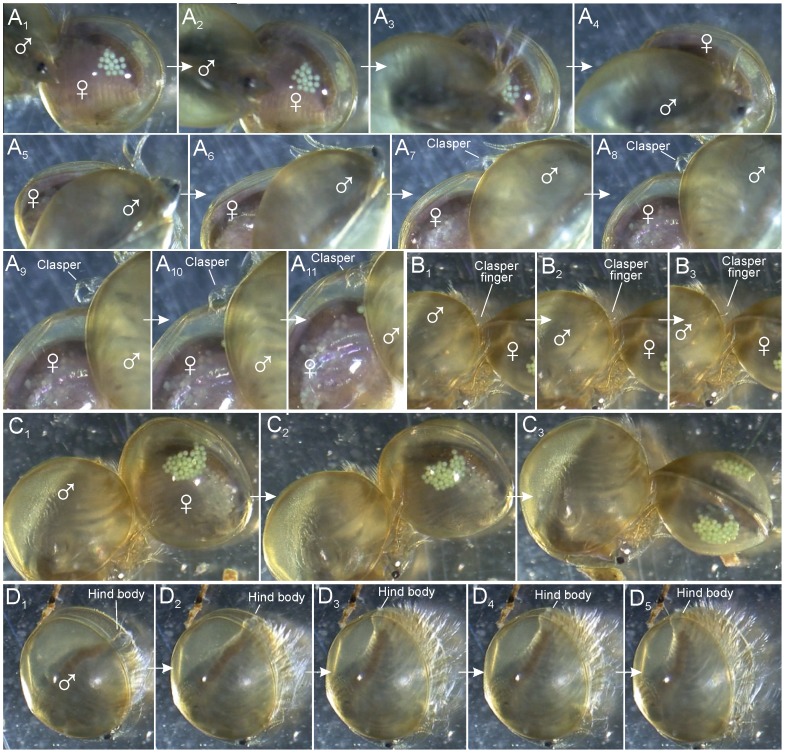
Male search behaviour and clasping attempts in *Lynceus brachyurus* (Laevicaudata). A_1–11_, video sequence showing male approaching female, passing over it, and extending clasper near female's carapace edge. Some frames have been left out. B_1–3_, male clasping female with one clasper (right side) and attempting to clasp with the other; movable finger is extended during this process. C_1–3_, male clasping female with one clasper (right side) attempting to insert the other clasper and in the process turn female so that the two animals are perpendicular of each other. D_1–5_, non-clasped male bending hind body and stretching it again similar to what takes place in a mating situation.

**Figure 6:**
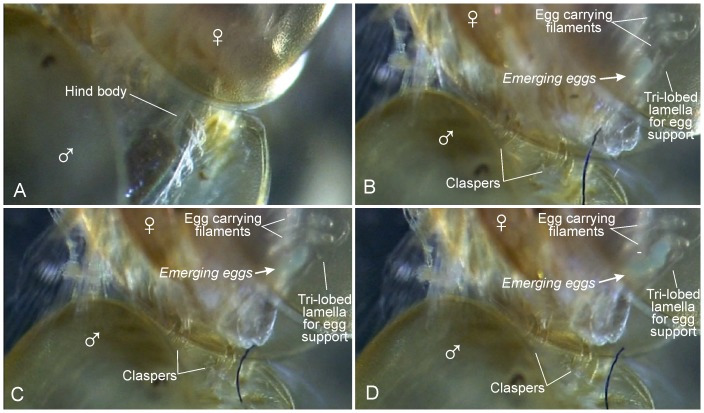
Successful mating and egg-laying in *Lynceus brachyurus* (Laevicaudata). A, male bending hind body deeply in between the valves and the limbs of a female while ‘brushing’ with its limbs. B–D, three different phases of early egg extrusion immediate after bending of male hind body seen in A.

**Figure 7:**
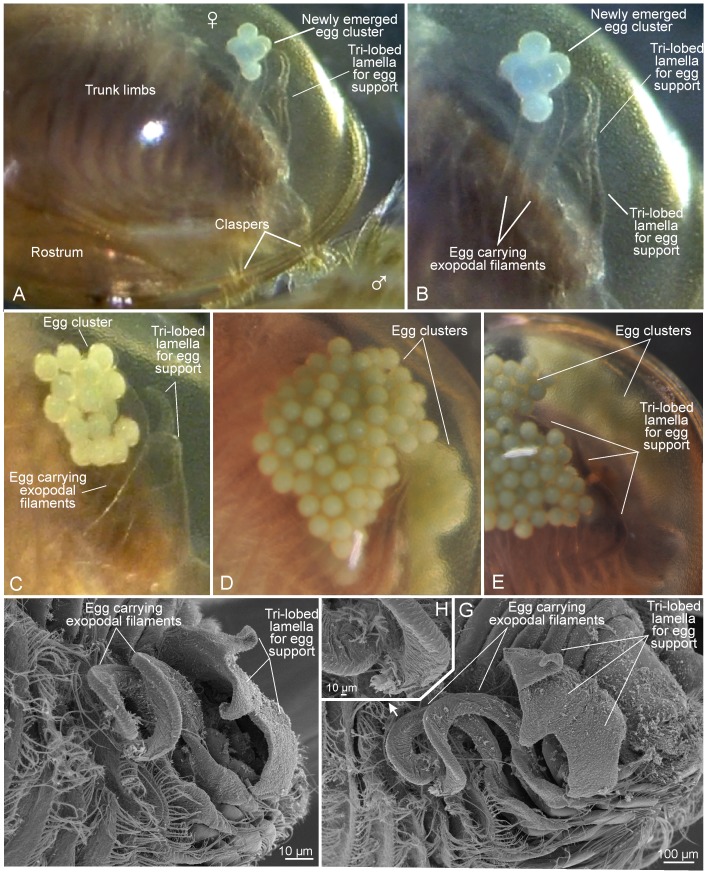
Egg clusters and egg carrying and egg supporting structures *Lynceus brachyurus* (Laevicaudata). A,B, female carrying small cluster of 6–7 eggs being attached to the tip of dorsal exopodal filaments of trunk limbs 9 and 10. C, female carrying cluster of about 16 eggs. D, female carrying clusters of at least 60 eggs. E, egg cluster supported by tri-lobed lamella. F, exopodal filaments for egg carrying and tri-lobed lamella for egg support. G, same as F from another angle. H, tips of exopodal filaments.

**Figure 8:**
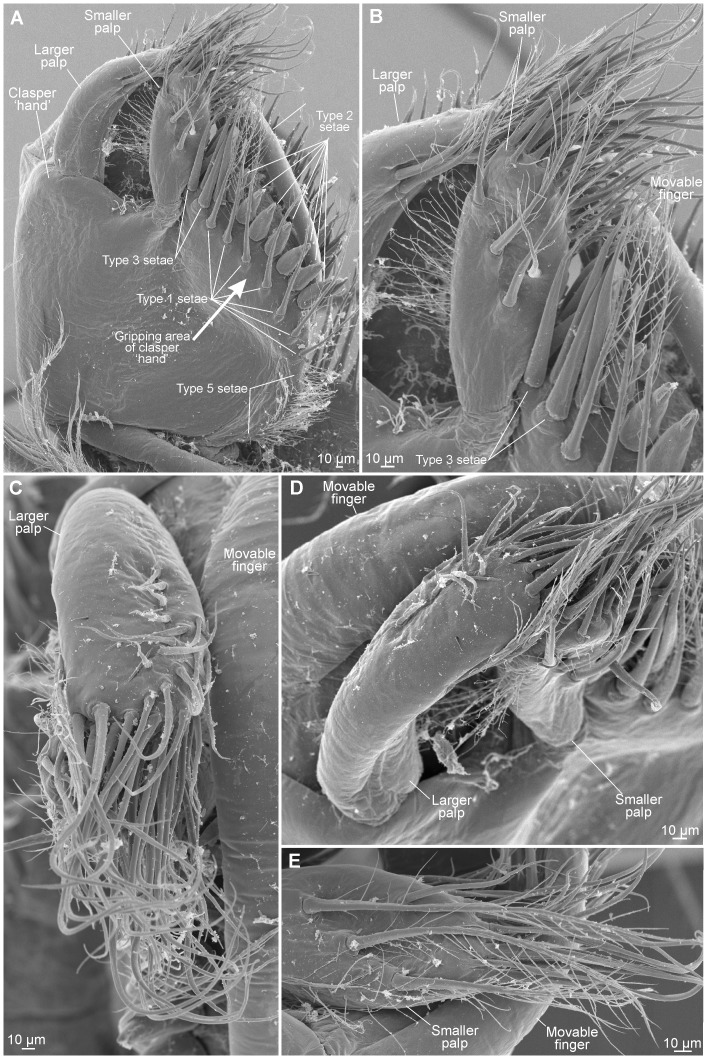
Right clasper of *Lynceus brachyurus* (Laevicaudata). A, clasper from posterior (median when attached). B, smaller palp. C, larger palp seen from apical. D, larger and smaller palp seen from apical. E, distal setation of smaller palp.

**Figure 9:**
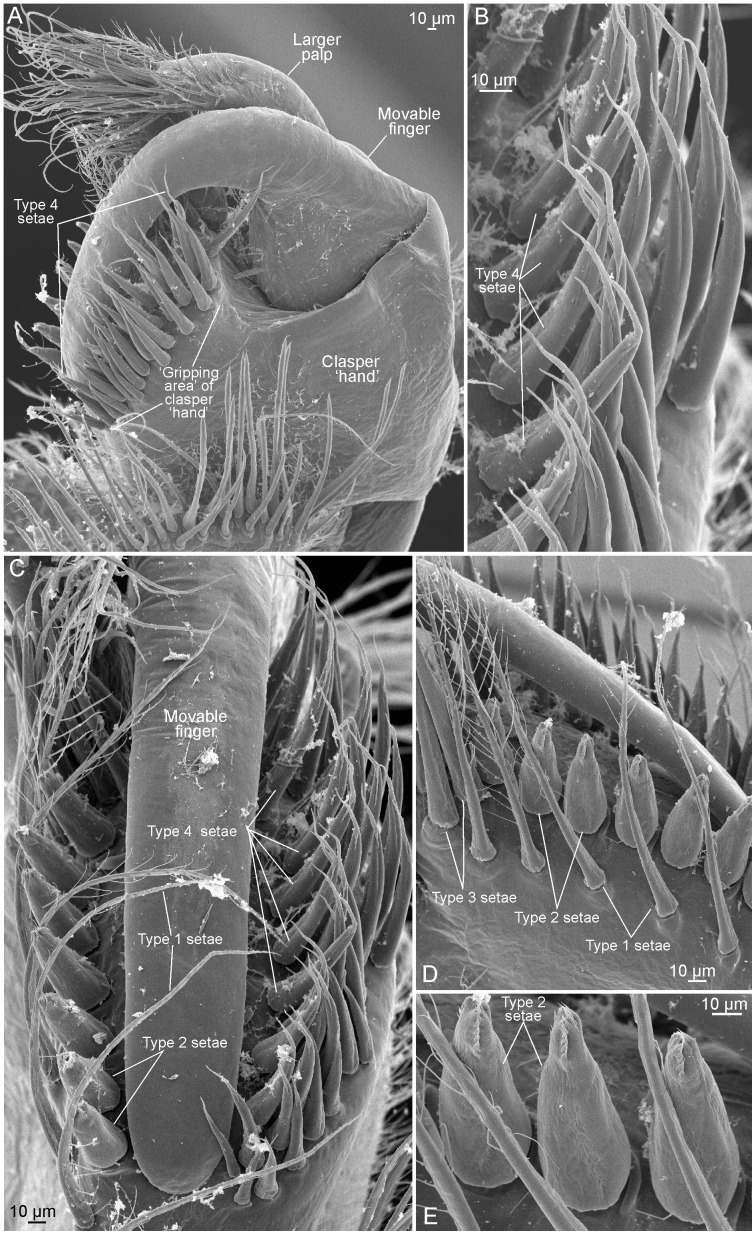
Right clasper of *Lynceus brachyurus* (Laevicaudata). A, clasper from anterior (lateral when attached to female). B, setae row on anterior side of movable finger. C, apical view on movable finger and setae rows on hand of both sides of movable finger. D, setae row on posterior side of movable finger. E, close-up of setae in D.

**Figure 10:**
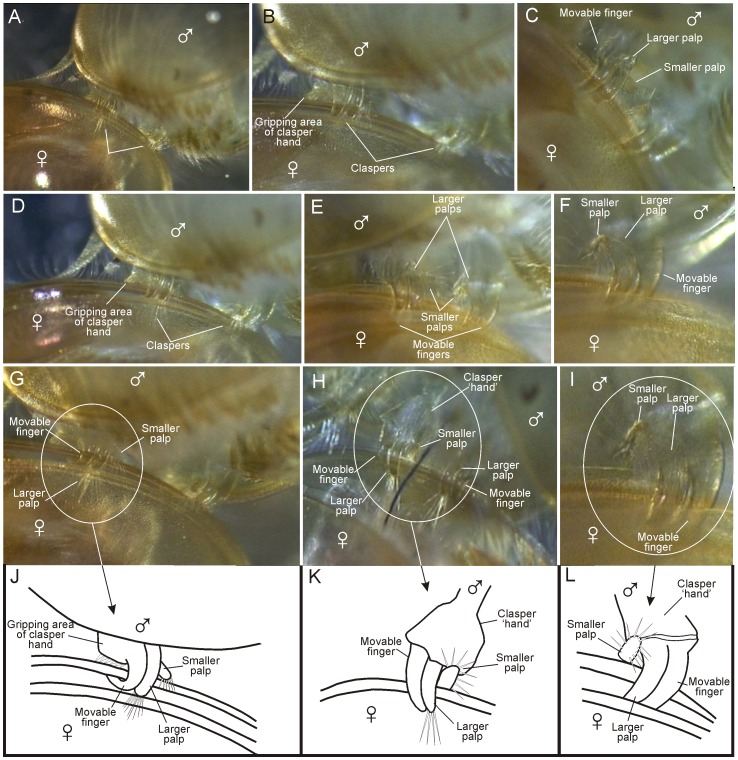
Position of clasper parts (‘hand’, movable finger, and palps) on female carapace edge of *Lynceus brachyurus* (Laevicaudata). A–I, close-ups of claspers attached to female carapace edge in various positions based of video recordings. J–L, line drawings of claspers in G–I.

**Figure 11:**
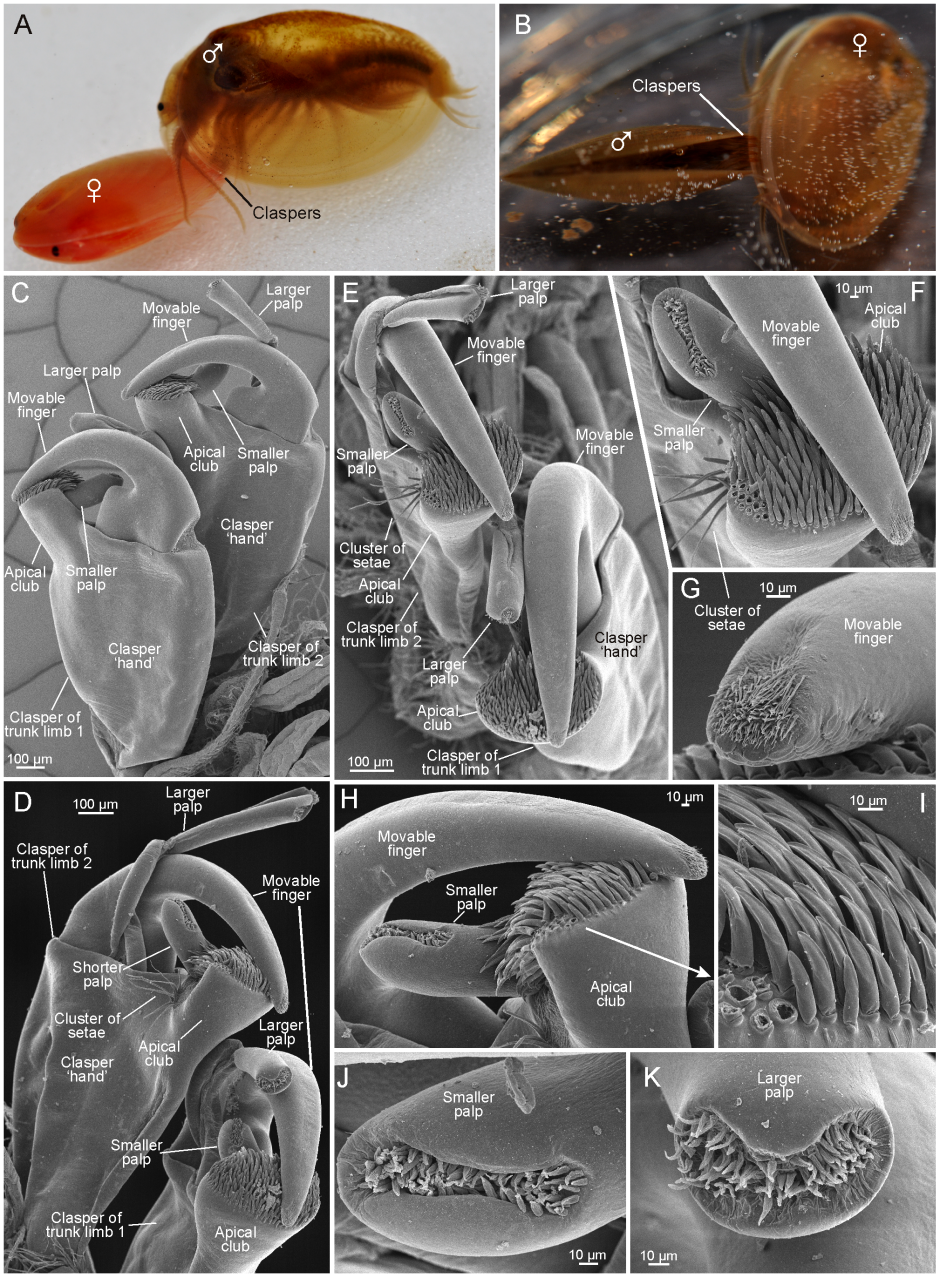
Clasping and clasper structures in *Cyzicus californicus* (Spinicaudata). A, B, male clasping female. C, pair of claspers (right side) of trunk limb 1 and 2 seen from anterior (lateral when attached to female). D, pair of claspers (right side) of trunk limbs 1 and 2 seen from posterior (median when attached to female). E, pair of claspers (right side) of trunk limbs 1 and 2 seen from apical. F, apical view of apical club, smaller palp, and movable finger of clasper of trunk 2 (right side). G, tip of movable finger of clasper of trunk limb 2 (right side). H, apical club, smaller palp, and movable finger of clasper of trunk limb 2 (right side). I, close-up on setation of apical club. J, distal setal field of smaller palp of smaller palp of clasper of trunk limb 2 (right side). K, distal setal field of larger palp of clasper of trunk limb 1 (right side).

*Lynceus brachyurus* O. F. Müller, 1776 (Laevicaudata), which reproduces gonochoristically (separate sexes) and is the only clam shrimp known in Denmark [18], [19] (Fig. 1B), was collected by JO from two different temporary ponds close to ‘Trepilelågen’ in Jægersborg Dyrehave (Deer Garden), around 10 km north of Copenhagen (Fig. 1A) in the spring (February–May) of 1994 and 2010. These ponds are two of the very few localities in Denmark where ‘large’ branchiopods are still present. The samples were collected using a plankton net (mesh size 63 µm) and brought to the laboratory of Zoological Museum and processed further as described below (Figs 1, 2, 3, 4, 5, 6, 7, 8, 9, 10). *Cyzicus californicus* (Packard, 1883) (Spinicaudata) was collected by JO and D. Christopher Rogers from Placer County, California, USA in April 2010 (Fig. 11A, B). A few habitus photos were taken with a standard mirror reflex camera (Nikon D90) using either a macro lens (*C. californicus*, Fig. 11A, B) or with the camera fitted to a stereoscopic microscope (*L. brachyurus*, Fig. 1B). Specimens of *L. brachyurus* and *C. californicus* were fixed/conserved in glutaraldehyde or 80% ethanol. They were dissected to expose the claspers using a stereoscopic microscope and dehydrated in a graded ethanol and acetone series. Afterwards the specimens were critical-point-dried using liquid carbon dioxide as the exchange medium, fixed on stubs with double-sided adhesive tape, and coated with metal. After these processes the specimens were observed and photographed with a scanning electron microscope (JEOL JSM-6335-F) with focus on the male claspers and their setae (Figs 1, 7, 8, 9, 11). Images were saved and processed digitally with various graphic programs such as CorelDraw, Photo Paint, Adobe Illustrator, and Adobe Photoshop. In 1994 and 2010 video sequences showing *Lynceus brachyurus*' mating behavior and male clasping organs in function were recorded using standard video equipment (from a course lab, 1994) or a Sony 3CCD ExwaveHAD (2010). The animals were filmed while kept in small petri dishes or larger glass bowls in the lab. The tape recordings were transformed from VHS and MiniDV to digital media using a JVC standard video recorder and the software Windows Movie Maker. Afterwards the videos sequences were observed several times with focus on general behavior, mating behavior, clasping of the female etc. and video sequences and still pictures were captured from the material using iMovie software. The frames were captured with a time resolution of 0,04s (25 frames/s) and pictures with a resolution of 640×480 pixels were further processed in Adobe Illustrator CS5.1 and Adobe Photoshop CS5 (Figs 2, 3, 5, 6, 7, 10). It has not been possible to add scale bars to the captured frames. Based on three of the captured pictures, clarifying drawings were made using overlay paper and a pencil (Fig. 10J–L).

## Results

The results are divided in four parts:

1. General mating behavior of *Lynceus brachyurus* (Laevicaudata) (based on videos)
2. External morphology of the claspers of *L. brachyurus* (based on SEM)
3. Function of the claspers of *L. brachyurus* based on close-up video observations
4. External morphology of the male claspers in *Cyzicus californicus* (Spinicaudata) with notes on functionalit

### General mating behaviour of *Lynceus brachyurus* (based on videos)

#### General

The animals were filmed while kept in small petri dishes and larger glass containers in the lab under conditions likely deviating from those of the natural habitat (e.g., probably higher specimen densities, higher temperatures,video S1). In total 154 minutes of video sequences has been examined. Clasping is occurring very frequently. 39 incidents of clasping of varying length are recorded on the videos but only in one case the mating was likely to have been successful (see below). Sometimes several couples are mating side by side in the same glass container.

#### Swimming

When not mating, or lying on the substrate, both males and females are swimming around in a more or less tumbling and diffuse way, turning around in the water, shifting direction very often. Most often swimming animals have the head oriented towards the swimming direction and their ventral side very close to (or touching) the bottom of the petri dish, and when encountering a piece of detritus or a conspecific animal they often make a short stop and approach the object with distal parts of their beating trunk limbs (video S1). Swimming is performed primarily by use of the second pair of antennae but also, to some extent, by the phyllopodous trunk limbs (also noted by [11], [12], [20]). Males are generally more active than females. Females often stay in the bottom of the petri dishes with the carapace valves completely closed while keeping the phyllopodous trunk limbs almost completely still. These differences between male and female movement patterns have not been quantified in further detail but similar trends have been reported in the androdioecious *Eulimnadia texana* (Spinicaudata) where males actively search their mates and thus swim faster than hermaphrodites [21].

#### Approaching the female, searching behavior, clasping position, and male/female size relation

Prior to clasping, it is observed that the male approaches the female's carapace either anteriorly or posteriorly (Fig. 5A). The male is apparently searching the edge of the carapace with his claspers for a little crack where he can insert the claspers (see video S1). Besides using the claspers to grasp the edge of one of the valves, the male also uses them to help spread the valves apart (see details below) (Fig. 5B). After having achieved a good hold on the edge of one of the valves, the male moves towards the female's ventral side where he makes a half rotation, so his head is placed over her carapace resulting in the male and female body being orientated perpendicular to each other (Figs 1B, 2A, B, 3A, B). Besides grasping the female with the claspers, the male places his rostrum and second pair of antennae on top of the female's carapace (Figs 2A, B). The rostrum of the male is abruptly truncate distally (in contrast to the long, pointed female rostrum) (Fig. 1C, G), which allows for close contact between the distal margin of the male rostrum and the female carapace. During pairing the couple normally lays still, but at certain points, the male starts to swim, pushing the female in front of him, briefly controlling the locomotion of the couple. In this situation the male lifts the antennae from the female's carapace and uses them for active swimming. In most of the observed pairings the male and female are about the same size (Fig. 4A).

#### Unsuccessful clasping

In the video material at least nine observations have been made of males approaching females in an unsuccessful attempt to clasp them. In several of the observations the male only attempts to clasp the female for a very short time and swims away again within seconds, but also more intense attempts have been observed, the longest lasting 35 seconds. During the approach, the male swims around the female apparently sensing the edge of the carapace with the claspers, trying to get a good hold on the valve (Fig. 5A). No signs of struggling by the female have been observed. The situation is more that the female lies inactively still, with the carapace valves closed, while the male spins around her trying to clasp her, but gives up after a short time and swims away.

#### Duration of clasping

We have little hard information on the video recordings on the duration of the claspings period such as the average time, since most of the clasping sessions are recorded without the beginning or the end. However, non-recorded observations in the lab indicate that the duration of each clasping typically is a few minutes. The longest recorded clasping lasted around 14 minutes and this was without the start and the end of the act, so the entire clasping session has probably lasted even longer. Most clasping sessions recorded lasted about a couple of minutes, but again most of them without the beginning and the end, or only one of them. There were three clasping sessions recorded from beginning to end lasting 70, 88, and 152 seconds, respectively.

#### Bending of hind body and brushing behavior of male

During clasping of the female, the male bends its hind body closer to the valves of the female's carapace and simultaneously perform some pulsating, metachronal movements of the limbs (Fig. 2C–F, 6A) (videos S1 and S3). The full sequence of events are typically as follows, but there are deviations from this standardized pattern (see below): (1) When the male has attached itself to the female's carapace margin, before bending its hind body, the trunk limbs perform slow metachronal movements interrupted once or twice per second by some rapid and forceful movements essentially ‘shaking’ all trunk limbs; typically about 5 ‘shakings’ of the trunk limbs are performed before the hind body is bend, but sometimes many more (15 has been observed); during this phase the female's carapace is typically almost closed while its trunk limbs are performing slow metachronal movements. (2) The bending of the hind body typically takes a couple of seconds; in this phase the entire body, including the trunk limbs, is bent ventrally to a position very close to the female's ventral carapace margin (Fig. 2D, F), and sometimes the hind body is placed between the valves (Fig. 6A); this is immediately followed by an intensive up-speeded ‘brushing’ by the trunk limbs where these are extended and sweep over both the female's carapace and trunk limbs. At the same time, when the ‘brushing’ is most intensive, the body of the male bends slightly dorsally again (same behavior seen in non-attached male in Fig. 5D) and since one of the female's carapace valves are ‘caught’ by some of small posterior trunk limbs of the male (Fig. 2F), or sometimes by the entire male hind body (Fig. 6A), this results in the two female carapace valves being spread slightly from each other; this ‘spreading’ of the females carapace can take place a number of times (3–4 times observed) exactly when the male's brushing is most intensive; when the male's ‘brushing’ becomes less intensive, the female carapace margins closes again more or less tight. During most mating attempts the female holds her carapace more or less closed, except when the valves are being spread apart by the male. The bending of the hind body in *Lynceus brachyurus* is rather similar to that described for *Eulimnadia texana* [7], [22], [23], but since it is much slower and less forceful compared to *E. texana*, the term ‘thrusting’, which was used for *E. texana*, has been avoided.

#### Male-male interactions with females

There are several observations of non-mating-males encountering males already attached to females. The approaching male either places himself on the male already present forming a ‘train’ (expression adopted from Knoll, 1995) (Fig. 3B), or to the opposing free valve of the female's carapace in such a way that a male is placed on both sides of her (Fig. 3A). Hind body bending and ‘brushing’ movements of the trunk limbs are performed by both males at the same time. At one occasion the approaching male succeeds in taking over the female but in most cases the approaching male leaves the female again (Fig. 4B). It is also seen that both males loosen their hold of the female resulting in the female swimming away. Several times it is seen that a male passes by a mating couple without starting any aggressive interaction.

#### Male-male interactions/encounters

Several times it is observed that males clasp other males behaving as if it is a female they clasp, i.e. they attach themselves to the male in the perpendicular way observed at male-female copulations and do ‘brushing’ movements with the trunk limbs (Fig. 3E, F) (video S2). Male-male couples are easily recognized by both having: a flat rostrum, no eggs, and most obvious: both having claspers. Another indicator is that the male ‘acting female’ is having its two valves significantly separated (Fig. 3E, F), which is different from a female where the valves are normally almost completely closed during mating (Fig. 1B, 3A, B). In one video sequence a third male appears and clasps the already clasped male resulting in three males attached in a triangle form (Fig. 3C, D). All of them moving actively, but with the one ‘acting female’ being most passive, causing a very confusing and disorientated swimming of the group. The two males attached to the carapace of the third male are doing ‘brushing’ movements with their trunk limbs as when they are clasping with a female (see above). There are also observations of a male clasping another male which is attached to a female (‘train’ position), and therefore already has the ventral side occupied; in such cases the new male does not attach himself ventrally on the other male, but instead posteriorly or anteriorly, either near the other males hind body or head, respectively (Fig. 3B).

Out of 39 observations of clasping sessions, 36 of them involve females. Only two are complete male-male interactions with two or three males connected (Fig. 4). In four other cases males are clasping other males (which are attached to females). In one video sequence there are as much as four animals attached to each other (3 males in a ‘train’ attached to a female).

#### Females: Egg extrusion and egg carrying

Most often the females that are clasped by males are carrying egg masses placed under the carapace, also prior to being clasped and ‘brushed’ (Figs 1B, 2A, B, 3A, B, 4D). Only in one case has egg extrusion been observed in a female while this was clasped and ‘brushed’ by a male (Fig. 6) (video S3). This female bore no egg masses prior to clasping. The process that lead to egg extrusion started with the male performing ‘brushing’ movements while bending its hind body ventrally and inserting it between the female's carapace valves; in this phase the phyllopodous limbs of the male and female interacted with each other (Fig. 6A). During this process the male's hind body was moved slowly dorsoventrally in about 10 seconds. Immediately after the male withdrew his hind body the female's body starts to move very slowly dorsoventrally while a greenish, amorphous egg-mass was extruded from a position laterally at the hind body immediate behind limbs 9 and 10 (probably from the basis of the 11^th^ trunk limb, see Linder 1945) (Fig. 6B–D). The same video show that the characteristically trilobed dorsal lamella on the side of the female's hind body, which are extensions from the female's somites (Fig. 7F–H), during egg-extrusion apparently guide the early string-like egg mass in the direction of the two exopodal filaments of trunk limbs 9 and 10 (Fig. 6B–D), the tips of which, with their clusters of small setae (Fig. 7H), are ready to collect the egg mass. Another video show the approximately same egg volume but now forming a cluster of 6–7 clearly defined eggs glued together by some unknown substance (Fig. 7A, B). This mass of eggs is now attached to the tips of the two exopodal filaments (details of comparable process provided for *Eulimnadia texana* by [24]). Yet another video show a female with an egg mass of about 16 eggs also attached to the tips of the two exopodal filaments (Fig. 7C). The filaments sometimes move the egg mass slightly around in different directions, but these movements are apparently connected to the movements of the entire limbs. In females carrying small egg masses (6–7 or 16 eggs) the trilobed dorsal lamellae are occasionally touching the exopodal filaments and/or the eggs and their function seems to be to keep the eggs in place. In females carrying a large egg mass (e.g., female in fig. 7D, E, with at least 60 eggs on each side) these are kept in place by the tri-lobed lamellae which are closely associated with the egg mass. When the female moves around or lie still, the egg mass, which consist of eggs ‘glued’ together, often perform small movements independent of the animal proper, showing that they are essentially suspended by the two exopodal filaments without firm attachment to other body parts.

### External morphology of the claspers of *Lynceus brachyurus*

The following description of male claspers is based primarily on observations made with scanning electron microscopy (SEM) (Figs 1D, 8, 9). Morphological terms used to describe the different structures are in general from following papers: [13], [20], [25], [26].

#### General shape and sexual dimorphism

*Lynceus brachyurus* is enclosed in a bivalved carapace with a hinge connecting the two valves dorsally (e.g., Fig. 1B, C). The valves are rounded in shape and are in lateral view almost as broad as long. The carapace lacks growth lines and has a markedly globular/spherical shape. The carapace can enclose the whole animal including its head. The size is approximately the same in both sexes. The head of both sexes is large, making up the bulk of the animal (Fig. 1C). The male has a distally truncate rostrum (Figs. 1C, 2A, B), whereas the female has a rostrum with a medial projection increasing its length and making it pointed distally (Fig. 1G). The rostrum, along with the male claspers and female eggs and egg-carrying/supporting appendages, are sexually dimorphic making the two sexes easy to distinguish.

#### Male claspers, general, movable ‘finger’

*Lynceus brachyurus* has the distal third of the first pair of trunk limbs modified into claspers (Fig. 1C, E). The right and left claspers are equal in size and shape (Fig. 1C). The claspers are circular in overall shape (Figs 8, 9) and each consist of a ‘hand’, a ‘movable finger’ and two palps, one larger and one smaller. The remaining part of the clasper limb, which is not treated further here, consists of a long limb corm with a distinctly pointed proximal endite and another more distal endite and laterally with a small sac-like epipod and a large lobate exopod (Fig.1E). Only the modified part of the trunk limbs are referred to as ‘claspers’ while the entire clasper-bearing limb is referred to as a ‘clasper limb’.

The movable finger is significantly longer than the larger palp (Figs 8D, 9A). It is curved, slender and arises at the outermost ( = lateral when limb is in resting position) distal end of the limb (Fig. 9A). Seen from the anterior side (when in resting position) of the clasper, it has a relatively broad base (Fig. 9A). The movable ‘finger’ is smooth, lacking any setae (Fig. 9C). In resting position the distal end of the movable ‘finger’ is closed against the opposing distal/apical part of the hand (Figs 8A, 9A). On this part of the palm there is a large cluster of diverse setae (described in the following sections). The movable ‘finger’ fits into a kind of groove with these setae on each side.

#### Setation of the clasper ‘hand’

Seen from the posterior side of the clasper (the palps' side) there are three types of setae on the distal part of the hand termed type 1, 2, and 3 (Figs 8, 9). Setae of type 1 are long, slender and with sparsely placed setulae extending in two rows from the shaft, making them plumose (Figs 8A, B, 9D). At high magnifications small denticle-like outgrowths are seen on the distal portion. There are seven of these setae arranged in a row and on the SEM photos they are bending over the movable finger (Fig. 9C). Setae of type 2 are spiniform, short, robust, and conical, with a significantly wide base and a rounded tip (Figs 8A, 9C–E). Apically they have two rows of small denticles (Fig. 9E). There are 7–8 of these setae placed in a sparse, longitudinal row and all are directed a bit away from the movable finger (Fig. 9C). Setae of type 3 are similar to the setae of type 1 in being long and slender, but they differ in being simple without any outgrowths and in being slightly stouter and shorter. There are 6–7 placed in a small and dense cluster near the base of the smaller palp (Fig. 8B).

Seen from the anterior side of the clasper (‘the finger's side’) there is only one kind of setae on the distal part of the palm placed in a dense row (Fig. 9A–C, setae type 4). They are of medium length, being shorter than the long slender setae on the posterior side of the finger but longer than the short stout setae also on the posterior side. The setae are bent with the concave side toward the movable finger so that the form of the setae fits around the shape of the finger. Distally these setae end in a characteristic bean sprout-like filamentous structure (Fig. 9B). A fifth kind of short, simple hair-like seta is present as a densely packed cluster located at the most proximal part of the clasper (Fig. 8A, setae type 5).

#### The palps

The two palps are both placed on the posterior side of the clasper (Fig. 8). When the claspers are actively clasping the carapace margin of another specimen, the entire clasper is turned 90° changing the functional position of the palps of each clasper so that they face towards each other medially. The larger palp is placed close to the base of the movable finger. On the specimens preserved and dried for SEM the larger palp is curved in the same direction as the movable finger. Seen from above (Fig. 8D), the larger palp is almost as wide as the finger, but at its base it is significantly more slender. The smaller palp is placed more medially at the clasper (Fig. 8A). It inserts on a small cuticular elevation. The tips of both palps are covered with long and slender simple setae (Fig. 8). The setae of the two palps are extending in the same direction resulting in their distal ends being mixed up with each other (in SEM prepared specimens) (Figs. 8D). The setae on the sides of the palps have a more diverse morphology. On the posterior side (when in clasping position) of both palps there are two (larger palp) or three (smaller palp) setae of more or less the same length as the setae extending from the tips, but they differ in bearing setules. Laterally the smaller palp has a row of several small filiform setae (Fig. 8B). Furthermore, the smaller palp has one short filiform seta close to the three longer plumose setae described above (Fig. 8B). The larger palp has, in addition to the setae at the tip and on the anterior side, shorter, simple setae extending from the dorsal side (Figs. 8D). These setae are placed in a kind of concavity and are of various lengths and pointing in different directions.

In summary it can be concluded that the smaller palp has four types of setae, whereas the larger palp has three.

### Function of the claspers of *L. brachyurus* based on close-up video observations

A brief overview of movement patterns of the claspers and their parts (movable ‘finger’, palps, and ‘hand’) is provided here (see video S3). A synthesis combining video and SEM information is given later (Discussion).

#### Claspers, attachment process

When the male is not attached to a female, the clasper limbs are orientated the same way as the more posterior limbs, with the tips of the movable ‘finger’ and the palps being directed medially or even posteriomedially (Fig. 1C, D). The male holds the claspers in an extended position when approaching a female, and one video recording shows how the ‘open’ side of the claspers is scanning a female's carapace near its margin probably to find a small opening where the movable finger of the clasper can be inserted (Fig. 5A) (see Discussion). Another video shows a situation where a male already clings to the female with one clasper (right side) and multiple times unsuccessfully attempts to insert the other clasper between the female's carapace valves which are closed at this stage. During this phase the left side clasper is turned 90° so that its median side (the clasper's ‘opening’ side) is facing the female's carapace margins. During each unsuccessful attempt to clasp the female, which takes place approximately once per second, the median side of the clasper (and its parts) is touching the females carapace margins and at least during some of these encounters, the movable finger is flexed open so that the female carapace margin would be easy to accommodate between clasper ‘palm’ and ‘finger’ (Fig. 5B). During each attempt to insert the clasper the male bends its body ventrally resulting in the male's posterior limbs and its hind body approaching the female's carapace valves (like during mating, see above), which probably plays a role in spreading the females carapace valves. Also during each attempt to clasp the female with the free male clasper, the male is capable of adjusting the orientation so that the female and male bodies lie perpendicular to each other (Fig. 5C), which is the mating position (see above), and which also facilitates insertion of the free clasper by simply bringing the female's carapace margin within reach of the clasper. Seemingly the truncate male rostrum plays a role in finding the right perpendicular orientation.

#### Position of the claspers during clasping

When in clasping position the male has placed his claspers around the margin of the female valve closest to him, with the ‘open’ part of the ‘hand’ in a forwardly directed position and with the movable ‘finger’ and the palps directed anteriorly (Fig. 10) (video S3). The distal parts of the movable finger and the larger palp are positioned on the inner side of the female's carapace (Fig. 10). The median part of the hand is placed on the outside of the female's carapace (Fig. 10B, D, J). Also the smaller palp is positioned on the outside but is bent in such a way that the tip and the attached setae are extending over the edge of the valve (e.g., Fig. 10F, I). In the clasping position, with both claspers placed firmly around the edge of the female's valve, the movable finger of each clasper is located laterally) and the smaller palp is located medially with the larger palp in between.

#### Orientation and movements of movable finger, palps, and ‘hand’ during clasping

The claspers are not kept entirely motionless while attached to the edge of the female's carapace. Some close-up videos show that there is no tight association between the distal part of the clasper hand (‘gripping area’) and the female carapace valves so that actually only the setae (setae of type 2 and 4) are touching the female's valve. In some videos it is observed that the male is repeatedly lifting and putting down the ‘gripping area’ of the clasper hand on the outer side of the female's carapace so that the setae (type 2) in this position are alternatingly bent and stretched. After having attached itself to the female's carapace margin, the male often changes position, which is achieved by slowly ‘walking’ with the claspers along the edge having one of the claspers attached at any time. During these minor sideways changes in position, the movable finger shows no distinct movement (but presumably it loosens its grip slightly). In contrast, the larger palp just next to the finger performs more or less constant movements without changing its basic position (bent around the female's carapace edge). The larger palp's distal long setae form a wide, mobile fan, often changing position during clasping (Fig. 10), and they respond rapidly on being occasionally touched by the female's moving limbs by bending away from these limbs. The shorter palp is placed immediate outside the carapace margin during clasping (Fig. 10). It performs small movements rather actively and the distal setae are spread out, some resting on the outer side of the valve while others reaches slightly around the margin.

### External morphology of the claspers of *Cyzicus californicus* (Spinicaudata) with notes on functionality

For comparative purposes we did a study on the clasper morphology of *Cyzicus californicus*, a representative of the Spinicaudata together with a few video-based observations.

#### The claspers, general, movable ‘finger’, and setation of clasper ‘hand’

*Cyzicus californicus* has the first two pairs of thoracopods modified into claspers which are used simultaneously when clasping the female (Fig. 11); the claspers of trunk limbs 1 are on the ‘outer side’ when attached to the female's carapace edge, the claspers of trunk limbs 2 on the ‘inner side’. All claspers are roughly of similar shape and size (Fig. 11C, D) and consist of a ‘hand’, a ‘movable finger’, and two palps, one larger and one smaller, respectively. The ‘hand’ is significantly elongated (Fig. 11C, D) and on its distal end, extends an ‘apical club’ against which the distal end of the movable finger closes during clasping (Figs 11E, F, H). The only apparent difference between claspers of trunk limbs 1 and 2 is a small cluster of setae at the base of the median side of the ‘apical club’ at the claspers of trunk limbs 2, being absent at the claspers of trunk limbs 1 (Fig. 11D, E, F). The movable finger is long, slender, and curved. It arises at the outermost distal end of the limb and has a significantly broad base (Fig. 11C). The finger closes against the apical club with its tip often extending over the edge of the apical club (Fig. 11E, F, H). The movable finger apex is covered with a dorsal dense cluster of short pointed setae (Fig. 11F, G). On the apex ventral side of the finger (the side facing the apical club) there are many scale-like outgrowths, slightly overlapping each other (Fig. 11H). The ‘hand’ setation is largely restricted to the apical club, which bears a dense cluster of relatively robust almost similar setae distally (Fig. 11F, H, I). All setae are smooth without any setules or outgrowths, and are moderately compressed along the distal half and have a sharp edge along the lateral sides. All are pointed the same direction towards the smaller palp and the tip of the movable ‘finger’.

#### The palps

Both palps are positioned on the functional posterior side of the clasper (small palp closest to midline) (Fig. 11D, E). The larger palp arises close to the basis of the movable finger. It is almost as long as the movable finger but more slender (Fig. 11D, E). A constriction/articulation divides the palp into a proximal and distal palpomere of approximately the same size. The proximal palpomere is twisted basally, which is probably partly an critical point drying artifact, but it shows that this part of the palp is more weakly sclerotizised and therefore more flexible. The larger palp is smooth except distally where it terminates in a cavity filled with a dense cluster of short, simple setae (Fig. 11K). The smaller palp arises at the posterior side of the apical club practically under the movable ‘finger’ and is directed towards its basis. The smaller palp is stout and unsegmented, terminating in an elongate, subdistal cavity, densely filled with small, simple setae (Fig. 11J).

## Discussion

### Mating behaviour of *Lynceus brachyurus*

#### Mate selection and mate guarding

Mating behavior has been studied in detail in some species of Spinicaudata [22], [23], [27], [28], [29], the most diverse group of clam shrimps. Little is known for representatives of Laevicaudata, another group of clam shrimps, which most likely is the sister group to all other clam shrimps and the Cladocera (grouped as Onychocaudata, see summary in [2]).

The overall impression is that *Lynceus brachyurus* (Laevicaudata) is immensely active when it comes to mating activity as are other clam shrimps (see [30]). Keeping *L. brachyurus* under laboratory conditions appears to have no negative impact on their mating frequency with several matings occurring next to each other (video S3). On the contrary, the mating frequency may be positively affected by the fact that the animals are placed side by side and that they therefore encounter each other more often than they would do in their natural habitats. The most common mating combination is male-female couples, but a number of male-male couples also occurred during the many hours of video recordings done in this work (Fig. 4C). It has not been tested whether the presence of male-male couples have been influenced by the artificial setup in the lab keeping in some cases several couples together for a long time in a relatively small volume of water, which may have concentrated eventual mating stimulating pheromones – if such exist. However, it should be noted that a very high percentage of male-male couples was found in the field in *Limnadia badia* [31].

The *L. brachyurus* male clasps the female with the first pair of trunk limbs (details discussed below). The two sexes are positioned perpendicularly to each other, which is also seen in other diplostracan taxa such as some spinicaudatans (e.g., *Cyzicus californicus*, fig. 11A, B) and cladocerans [29], but not in *Paralimnadia* and *Limnadopsis* where the female and male line up in the same plane [32]. Besides grasping the female using the claspers, the male uses his rostrum and second pair of antennae on top of the female's carapace (Fig. 2A, B). The male's rostrum is truncate distally (Fig. 1C) (in contrast to the medially pointed rostrum of the female, Fig. 1G), which facilitates its placement closely associated with the female's carapace during mating. The position of the antennae on top of the female's carapace is probably to assist in keeping the male in position. In the light of this knowledge, the mating couple of *L. gracilicornis* depicted in [26] is possibly drawn in an atypical mating position.

We found no fixed size-relation between males-females of mating couples, so there is probably no selection on preferred size by either the males or the females. In most cases the female is either the same size or only slightly larger than the male (Fig. 4A). However, in two cases the male is significantly larger than the female. The apparent lack of size-based mating preference is in accordance with studies on *Eulimnadia texana* where it has been shown that neither hermaphrodites nor males show any sign of mating preference based upon size [28]. However, in the same species, it has been found that larger males were advantageous in cases of ‘intersexual conflicts’ and resulted in longer guarding times [30], [33]. Observations of *E. texana*, showed that when hermaphrodites (who can self fertilize their own clutch of eggs) are not interested in mating, because they are not receptive, they struggle against the male so that the male releases his hold and no mating occurs [22]. In this study, females of *L. brachyurus* show no struggling against the male. Instead the female lies inactively still, with the carapace valves closed, while the male spins around her trying to clasp her (e.g., Fig. 5A). Sometimes the male gives up and swims away, which may be because the female does not open the carapace enough to make the male able to place his claspers around the edge of the valve. Whether there is a ‘choice’ from the female's side regarding when to open the valves, or whether it is entirely up to the male to force the female's valves apart is not known.

Aggressive encounters between males of *E. texana* has been observed, and it was most often seen that the larger male would be the winner of these encounters and take over the female from a smaller male already clasping a female [28]. In *Limnadia badia*, it was found that when two males clasp a female the kick each other vigorously, attempting to dislodge the rival, they found no significant size differences between ‘loser’ and ‘winner’ males [27]. In *L. brachyurus* this pattern seems different as the approaching male most often does not take over mating, and also we observed no fighting between males as reported for *L. badia*.

#### Mating strategy in Lynceus brachyurus

Clasping of *E. texana* is described as involving (precopulatory) mate guarding, which allows males to be present near hermaphrodites/females when they are receptive (after molt), and at the same time prevent other males from mating [7], [22], [23], [27], [28], [29], [31], [33]. In *E. texana* the male carries the hermaphrodite for an extended period of time (from minutes to hours) and the male clasps the hermaphrodite so forcefully with its two pairs of claspers that the couple can be lifted out of the water without the male's grip being lost [23], which is also seen in *Paralimnadia badia* [31]. We have not in detail examined to which extent precopulatory mate guarding is present in *L. brachyurus*, since this would require recording of the overall clasping time in a larger number of mating pairs, and relating this with the actual time for fertilization (probably when the eggs are extruded, see below). However, mate guarding, if present, at least appears to be less pronounced in *L. brachyurus* since average clasping time is shorter and less forceful, and the male in general exerts much less control over the female than what was reported in *E. texana*. It seems that the mating strategy of males of *L. brachyurus* is to attempt to mate with as many females as possible in order to increase the chances of fertilizing the eggs of the females. Males are seen performing ‘brushing’ movements no matter whether they are attached to females or to other males (the latter shown in Fig. 3). Most of the amplexed females carry full egg clusters of varying sizes (Figs 2A, B, 3A, B, 5C, 7), only in two cases was mating between a male and a female without eggs seen (Figs 4D, 6). This does not necessarily mean that males prefer mating with egg-bearing females, but may simply reflect that egg-bearing females were more abundantly around; no experiments were designed for testing this. When two males are attached to the same female – one male on each side of the female's carapace (Fig. 3C, D) – both males can be seen ‘brushing’ at the same time. In one of the videos it is observed that the same female is getting clasped twice (probably by the same male). What is the advantage clasping and ‘brushing’ other males and females with eggs that are probably already fertilized? Perhaps none. It may be that the males simply cannot tell the sexes apart or distinguish between females with fertilized or unfertilized eggs, at least not prior to clasping and the initial phase of the ‘brushing’. It has been suggested that non-receptive ( = after egg extrusion) hermaphrodites of *E. texana* may release a chemical non-receptive cue [28]. Furthermore, it has been found that males of *E. texana* are able to assess receptivity of the hermaphrodites after physical contacts which suggest that chemical communications (possibly ecdysal hormones) may be important [7]. Currently there is no behavioral data for the mating of *L. brachyurus* to suggest that early physical contact or chemical cues play a role in this species, but this is certainly possible and specific experiments should be designed for this purpose.

#### Male ‘brushing’ and female egg extrusion

In all the observed clasping events, the male of Lynceus brachyurus is doing ‘brushing’ movements moving the lower portion of his body up between (or near to) the two valves of the female's carapace (Figs 2C–F, 6A), a behaviour that must be assumed to play a role in sperm transfer and egg fertilization (videos S1 and S3). Several authors have used the used the term ‘thrusting behavior’ for a comparable pattern [7], [22], [23] (see Results). Exactly when and how sperm transfer occurs, and at what time fertilization takes place in Lynceus has not been directly observed in this study. Indeed, this is unknown with certainty for any clam shrimp species. We observed one case in Lynceus where egg extrusion followed immediately after a male had ‘brushed’ a female very intensively, and probably this part of the ‘brushing’ sequence in Lynceus is equivalent to the thrusting behavior of ‘type 2’ as it was described from Eulimnadia texana [23]. After the male had thrust its hind body deeply in between the carapace valves of the female, a greenish, amorphous egg-mass appeared dorsolaterally and was collected by the egg carriers (Fig. 6B-D) (video S3) (comparable process described for E. texana [24]). The fact that egg extrusion has only been observed once in several hours video observations of Lynceus pairings suggest that this occurs only rarely. For E. texana, Weeks et al. [23], building on [22], found experimental evidence that fertilization of hermaphrodites by males (outcrossing) is external and suggested that there is a narrow ‘window of opportunity’ directly after the eggs has been extruded where male sperm can invade the egg shell. We do not have comparable experimental data for Lynceus, but the single observation of correspondence in timing between particularly intense and deep bending movements of the male hind body followed by female egg extrusion (Fig. 6, see details in Results) seems also to suggest external fertilization in this species and that it occurs in relation to egg extrusion. Most likely egg extrusion in Lynceus is initiated by the male's mating movements e.g., the characteristic ‘brushing’ movements. Video recordings show that the eggs of the female emerge laterally at the body immediate below and slightly posterior to the exopodal prolongations of trunk limbs 9 and 10, by which they get caught immediate after release (Fig. 6B–D). This conforms to earlier findings of the female genital openings to be at the proximal parts of the 11^th^ pair of limbs [34]. Convincing evidence for the position of the male genital openings to be on both sides of the anal openings at the end of the body has also been provided [34]. These positions of the female and male genital openings fits well with the observed pattern during mating, since these exact body parts of the female and the male are brought very close together and seem to have physical contact, especially during the single observation of a clasping-brushing event leading to egg extrusion (Fig. 6). A mechanism of timing must be present to coordinate male sperm transfer with female egg extrusion to avoid waste of both leading to unsuccessful mating. While there is much evidence of complicated physical contact between the female and male during mating (e.g., male ‘brushing’), which may play a coordinating role, we did not investigate the possibility of chemical communication. Perhaps the extended male ‘brushing’ and repeated attempts to insert the male's hind body between the female's carapace valves serves as preparation of both the male and the female for mating leading ultimately to male sperm transfer. Female egg extrusion occurs immediately after the assumed sperm transfer (Fig. 6) so it could be prompted chemically. The spermatozoans of L. brachyurus are known to be of the typical branchiopod amoeboid type [15], but no information on whether the male attach spermatozans in the form of spermatophores to the female or they are shed freely within the carapace chamber of the female is available. Experiments for E. texana suggest that fertilization occurs in relation to the male thrusting the female, indicating that no spermatophore is involved [23]. However, this is in conflict with [35] where, in a large proportion of hermaphrodites (termed females in [35]) orange structures interpreted as spermatophores were found attached to the limbs (see [36], and critical comment by [24]). For L. brachyurus we noted that opposite the clasped males, clasped females tend to keep their carapace valves closed during the clasping male's mating activities except for the brief periods when the male is inserting his hind body between the valves. This tendency of keeping the carapace valves closed could be interpreted as a female adaptation to keep eventually released spermatozoa of the male in the vicinity of the newly extruded eggs, something irrelevant of clasped males.

Observations of egg-laying in *Lynceus brachyurus* showed that not all eggs are extruded at the same time. For example, in the single case were egg extrusion was directly observed following male mating activities only 7–8 eggs emerged before they were collected by the exopodal filaments at trunk limbs 9 and 10 (Fig. 7A, B). Females with a varying number of eggs on each side were observed (e.g., 16–18, figs 5A, 7C). Therefore, to obtain the large egg clusters carried by most females (more than 50, figs 1A, 2A, B, 3A, B, 5C, 7D, E) more than one egg extrusion is necessary. If indeed fertilization occurs externally immediately after egg extrusion in *L. brachyurus*, as inferred above and as experimentally suggested for *E. texana* [23], then it implies that only a fraction of the eggs in the egg cluster are fertilized at the same time and that different eggs in the egg cluster potentially are fertilized by different males. This would be another explanation of the immense mating activity seen in *Lynceus* where males very often attempts to mate with females that already carries eggs. In copepods, where potential advantages of multiple mating have long been discussed (e.g., [37]), partial fertilizations of egg clutches have now been reported for *Temora longicornis* (Müller, 1792) [38]. It has been suggested that polyandry (females mated by many males) play a role when females do not have reliable criteria for discriminating male genotypes, and that it has advantages in environments that vary over time (e.g., temporary pools) by increasing the genetic diversity of the offspring [39], [40].

### Functional morphology of the claspers of *Lynceus brachyurus* with notes on spinicaudatans

The claspers of *Lynceus brachyurus* and other clam shrimps have received attention from a comparative morphological (phylogenetic) perspective [13], [14], but no detailed functional analysis has been carried out, so except for the fact that they are used for clasping the females during pairing, no functional aspects are known. The presence of claspers on the anterior trunk limbs has been suggested as a synapomorphy of clam shrimps and water fleas [4], [13]. However, the homologies at a more detailed level between the rather similar claspers (see Introduction) of the two major clam shrimp groups, Laevicaudata (e.g., *Lynceus brachyurus*) and Spinicaudata are still open to discussion. The claspers in Laevicaudata and Spinicaudata have been suggested to be only partial homologous [13]. The ‘hand’ – the structure opposing the ‘movable finger’ during clasping – and the two palps (small and large) were considered derived in two different ways in the two groups, while the movable ‘finger’ was considered homologous. In this light, a study of functional aspects of the claspers is interesting. For example, is the partly non-homology expressed in the function of the various clasper parts? As a first step in such a comparison of clasper functionality in clam shrimps, an analysis of the functional morphology of the clasper during mating in *L. brachyurus* is provided here and compared with the limited information available for spinicaudatans.

In resting position the clasper limbs in *Lynceus brachyurus* are held with a similar orientation as the following phyllopodous legs, with the palps and the movable finger directed medially or even postero-medially (Fig. 1C, D). But when the male is approaching a female in order to clasp it, the movable finger and the endites are turned 90° which enables the clasper to clasp the female carapace margin (see position in Fig. 10). When the male claspers have clasped the female's carapace valves, the distal part of the clasper ‘hand’ and the proximal parts of the movable finger and the larger palp are on the outer side of the valve, while the distal parts of the movable finger and the larger palp are on the inner side (Fig. 10). The smaller palp is bent in such a way that the tip and the attached setae are extending over the edge of the valve (Fig. 10E–L). Often the male of *L. brachyurus* shifts its position on the female's carapace margin by ‘walking’ with the claspers releasing the grip of only one clasper at the time. In some species (e.g., *C. californicus*, Fig. 11A, B, and *C* *. grubei*, see [29]), males are attached to the female (or the hermaphrodite) carapace margins in essentially the same perpendicular way as in *L. brachyurus*, except that two pairs of claspers are used simultaneously with the claspers of trunk limbs 1 being at the ‘outer side’ when attached to the female's carapace margin. In contrast, *Limnadopsis* and *Paralimnadia* males amplex the posterior margins of the female carapace, with their body in line with the females (not perpendicularly) during clasping [32].

The parts of the clasper in *L. brachyurus* responsible for clinging to the female's carapace margin is the movable finger and the distal part of the ‘hand’ which are pressed together on both sides of the edge valve. Strong musculature supports the movable finger which testifies its function as the active part of the clasper during clasping (1F). The movable finger in *L. brachyurus* has no scales or setation which is in contrast to many spinicaudatans where scales (e.g., *C. californicus*, Fig. 11G) or even a sucker-like structure (in many limnadiids, e.g. [32], [41]) are present, or to Cyclestherida where the movable finger has a row or long setae [13]. The scales in *C. californicus* are placed subdistally on the ‘fingertip’ on the side facing the inner side of the carapace, and probably serves to enhance surface tension and get a better grip. The elaborate scales/spines in this region of the finger in various species of *Limnadopsis* have been suggested to possibly play a role in mate recognition [42], but may alternatively be interpreted as hooks assisting in separating the female carapace valves from each other prior to clasping. The distal part of the ‘hand’ in *L. brachyurus* has a diverse setation which probable plays both a mechanical and sensory role, the details of which have not been directly studied here, and which will probably prove technically very difficult to do. However, we assume some of the longest setae (type 1, Fig. 9C) of the hand may play a role in detecting the precise position of the female's carapace margin prior to clasping. The row of characteristic setae (type 4) on the outer side of the movable finger (when in clasping position), all of which ends in a slender, filiform soft part (Fig. 9A–C), are probably also sensory. Some close-up videos show that there is no tight association between distal part of the clasper hand and the female carapace valve while clasping. It actually appears as though only the setal row (type 4) are touching the female. During the slight movements of the clasper, the distal part of the ‘hand’ is occasionally lifted from the female's carapace resulting in the setae in this row bending back to a more neutral position. This change in setae orientation depending on the distance between a part of the clasper and the female's carapace valve suggest that they could be important in detecting how firm the grip around the carapace margin is by flex reception and thereby providing information for controlling clasping strength. We have no video-observations on the medially placed setae row since it is ‘hidden’ by the movable finger in all recordings (type 2, Figs 8A, 9D, E). They are shorter and more robust distally with denticles that may provide the clasper with a firm grip around the female clasper margin, but may also play some kind of role in detecting the clasping pressure. Taken together, the organization of the setae in two distinct rows on each side of the movable finger, with the setae in each row pointing away from the midline of the clasper (Fig. 9C) probably also have a mechanical function in providing some elasticity to the clasping process since the setae of the rows will offer more and more resistance as they are pressed against the female's carapace. In contrast, the distal part of the clasper ‘hand’ in spinicaudatans largely have confined the setation to the so-called ‘apical club’ (e.g., Fig. 11), which is the part of the clasper hand opposing the female carapace from the outside during clasping. The cluster of setae at the apical club with their bended and flattened appearance probably provide mechanical resistance during clasping and sense pressure force, similar to what was suggested above for setae types 2 and 4 distally at the clasper hand of *Lynceus*.

During clasping in *L. brachyurus* the two palps are placed in a very characteristic way in relation to the carapace margin and they certainly have sensory functions (Fig. 10). The larger palp is slightly shorter than the movable finger and is placed immediately next to this and is bent the same way around the female's carapace margin. It performs small movements but maintains its bent position during all observed matings. Distally it carries a cluster of long setae, which during clasping most often are spread out as a fan (Fig. 10G, H, J, K). When the female performs abrupt movements within its carapace, this setal fan responds rapidly and bends closer to the inner side of the female's carapace valve. Since the setal fan of the larger palp is directed in between the female's carapace valves we suggest that the male is able to detect the position of the female, and thereby better choose the right clasping position which increases the chances of successful mating. We have observed several occasions where the male first attaches itself in a posterior or anterior position at the female's carapace, followed by slow changes in position to a more ventral position. Another possible function of the distal setal fan of the larger palp would be for the male to detect when the female carapace is open prior to the onset of mating movements. The setal fan of the larger palp may also play a sensory role earlier in the mating process when the male approaches the female with extended clasper legs attempting to find an opening between the female's carapace valves where the movable finger can be inserted (Fig. 5A, B). In spinicaudatans the position of the longer palp during mating is known for a couple of species (e.g., *C. tetracerus* (Krynicki, 1830), see [43]), and, as in *Lynceus*, it is bent around the female (or hermaphrodite) carapace margin at the inner side, following its curvature suggesting a sensory function similar to *L. brachyurus*. However, detailed knowledge of the role of the longer palp prior to or during clasping remains to be studied for species of the Spinicaudata. The smaller palp in *L. brachyurus* is placed immediate outside the carapace margin during clasping. It is moved more around than the longer palp and the distal setae are spread out, some resting on the outer side of the valve while others reaches slightly around the margin. The setation of the smaller palp may play a sensory role in detecting the exact position of the carapace margin of the female in relation to the clasper, or may provide information on the level of clasper pressure provided by the movable finger. The smaller palp in spinicaudatans in general has a quite different morphology from that of *L. brachyurus* with setation confined only to a distal groove and shown for *C. californicus*. In *C. californicus* the smaller palp is placed closer to the tip of the finger at the basis of the apical club, but the palp is directed towards the midline of the clasper hand so that its setose tip are in a position very similar to that of the smaller palp of *L. brachyurus*, suggesting that also here it may play a role in sensing the position of the clasper relative to the female's carapace margin. It is also possible that the setae of the palps are chemosensory and involved in detecting whether female egg extrusion is near possibly requiring physical between the sexes as it was found in *Eulimnadia texana* (see [7]).

The overall impression is that the claspers of spinicaudatans in general are adapted for getting a stronger hold of the female (or hermaphrodite) than is the case for laevicaudatans (at least *L. brachyurus*). This is suggested first of all by the presence of two pairs of claspers in spinicaudatans contrary to only one pair, but also by the combined presence of an apical club distally at the clasper hand and distal ornamentation (scales) of the clasper finger. Brief observations on the mating behavior of *Cyzicus californicus* indeed shows that the male are attached very strongly to the female (couple can be moved around with forceps), something which may be related to extended mate guarding being a part of the mating process in these species.

If suggestions concerning homologies of laevicaudatan and spinicaudatan claspers are correct, then the movable fingers are homologus and the palps partly homologous, while the ‘hand’ of the claspers, including the specific point of the hand that opposes the carapace externally (the ‘gripping area’), are non-homologous [13]. This is at least not contradicted by the present study, in which the examined laevicaudatan and spinicaudatan species show a fundamentally different type of setation of the parts of the clasper hands opposing the tip of the movable finger when clasping, despite having similar functions.

## Conclusions

This study presents a detailed video-based study of mating behavior and functional morphology of the male clasper of *Lynceus brachyurus* (Laevicaudata), a phylogenetically pivotal taxon of clam shrimps. *L. brachyurus* exhibits an immense mating activity clasping females and other males, as well as females with and without eggs. We suggest that this is a strategy to fertilize as many eggs as possible and that mate guarding is not as pronounced in *L. brachyurus* as in various spinicaudatans. It has been possible to link the morphology of many structures to their function based on the video recordings of live material. For example, during the male's mating movements, the hind body and its shorter limbs assist in spreading the female's valves apart. Females and males respond different when being clasped and ‘brushed’ by a male: females tend to close their carapace after each mating attempt, while males keep them apart, which may be an attempt from the females to keep eventual sperm inside the carapace cavity. One case of egg extrusion was observed and is shown that tri-lobed lamellae at the sides of the females hind body assist in guiding the newly extruded egg mass to the tip of the egg carriers (modified exopods) by which they are carried under the carapace. Most focus of this study was on the morphology and functionality of the male claspers. Based on close-up video recordings of male claspers while attached to the female's carapace edge, the natural positions of the various clasper parts are revealed. The movable finger is the clasper part actually taking care of the clasping. The two palps are both placed in a very characteristic way at the carapace edge and, based on their movement pattern and setation, are certainly sensory playing a role in operating the clasper at the carapace margin.

## Supporting Information

Video S1 **Male of** ***Lynceus brachuyrus*** **clasped to female while he gets clasped by another male.**
(M4V)

Video S2 **Male with one clasper (left) attached to female either trying to free himself or to get a grip with the other clasper.**
(M4V)

Video S3 **Male attached to female's carapace showing how the clasper parts are orientated at the carapace margin; later the male inserts his hind body between the female's carapace halves which is followed by female egg extrusion.**
(M4V)
